# Regulation of Lipolysis and Adipose Tissue Signaling during Acute Endotoxin-Induced Inflammation: A Human Randomized Crossover Trial

**DOI:** 10.1371/journal.pone.0162167

**Published:** 2016-09-14

**Authors:** Nikolaj Rittig, Ermina Bach, Henrik Holm Thomsen, Steen Bønlykke Pedersen, Thomas Sava Nielsen, Jens O. Jørgensen, Niels Jessen, Niels Møller

**Affiliations:** 1 Department of Internal Medicne and Endocrinology (MEA) and Medical Research Laboratory, Aarhus University Hospital, Nørrebrogade 44, 8000, Aarhus, C, Denmark; 2 Research Laboratory for Biochemical Pathology, Institute for Clinical Medicine, Aarhus University Hospital, Nørrebrogade 44, 8000, Aarhus, C, Denmark; 3 The Novo Nordisk Foundation Center for Basic Metabolic Research, Faculty of Health and Medical Sciences, University of Copenhagen, Blegdamsvej 3b, 6.6.30, DK-2200 N, Copenhagen, Denmark; Universita degli Studi di Milano, ITALY

## Abstract

**Background:**

Lipolysis is accelerated during the acute phase of inflammation, a process being regulated by pro-inflammatory cytokines (e.g. TNF-α), stress-hormones, and insulin. The intracellular mechanisms remain elusive and we therefore measured pro- and anti-lipolytic signaling pathways in adipocytes after *in vivo* endotoxin exposure.

**Methods:**

Eight healthy, lean, male subjects were investigated using a randomized cross over trial with two interventions: i) bolus injection of saline (Placebo) and ii) bolus injection of lipopolysaccharide endotoxin (LPS). A ^3^H-palmitate tracer was used to measure palmitate rate of appearance (*Ra*_palmitate_) and indirect calorimetry was performed to measure energy expenditures and lipid oxidation rates. A subcutaneous abdominal fat biopsy was obtained during both interventions and subjected to western blotting and qPCR quantifications.

**Results:**

LPS caused a mean increase in serum free fatty acids (FFA) concentrations of 90% (CI-95%: 37–142, p = 0.005), a median increase in *Ra*_palmitate_ of 117% (CI-95%: 77–166, p<0.001), a mean increase in lipid oxidation of 49% (CI-95%: 1–96, p = 0.047), and a median increase in energy expenditure of 28% (CI-95%: 16–42, p = 0.001) compared with Placebo. These effects were associated with increased phosphorylation of hormone sensitive lipase (pHSL) at ser^650^ in adipose tissue (p = 0.03), a trend towards elevated pHSL at ser^552^ (p = 0.09) and cAMP-dependent protein kinase A (PKA) phosphorylation of perilipin 1 (PLIN1) (p = 0.09). Phosphatase and tensin homolog (PTEN) also tended to increase (p = 0.08) while phosphorylation of Akt at Thr^308^ tended to decrease (p = 0.09) during LPS compared with Placebo. There was no difference between protein or mRNA expression of ATGL, G0S2, and CGI-58.

**Conclusion:**

LPS stimulated lipolysis in adipose tissue and is associated with increased pHSL and signs of increased PLIN1 phosphorylation combined with a trend toward decreased insulin signaling. The combination of these mechanisms appear to be the driving forces behind the increased lipolysis observed in the early stages of acute inflammation and sepsis.

**Trial Registration:**

ClinicalTrials.gov NCT01705782

## Introduction

Administration of bacterial endotoxin is widely used as a model of acute inflammation and sepsis [1,2]. Energy demands increase during the initial phase of sepsis and is mainly provided by lipid mobilization and oxidation [3,4]. Storage of lipids in adipose tissue constitutes the largest endogenous energy supply and lipolysis therefore becomes essential during conditions with acute increased energy demands [5]. Notably, lipolysis is elevated in septic patients during the first days of hospital admission [6–8]. Despite this, knowledge about the regulation of lipolysis in human adipose tissue during sepsis and acute inflammation is sparse [9].

Lipolysis is rapidly and distinctly stimulated by epinephrine, but other hormones such as cortisol and growth hormone (GH) also all contribute and increase acutely in response to sepsis [10]. In addition, bacterial products (such as endotoxins) cause increased concentrations of proinflammatory cytokines (e.g. TNF-α), which are known to have lipolytic actions [11–13]. In contrary, insulin remains the principal anti-lipolytic agent, although ketone bodies also suppress lipolysis [14,15].

As reviewed in detail by others [16,17], lipolysis is tightly regulated through adipose triglyceride lipase (ATGL) and hormone sensitive lipase (HSL), of which ATGL is the initial rate-limiting enzyme in the conversion of triacylglycerol (TAG) into glycerol and free fatty acids (FFA). HSL activity is regulated through phosphorylation on multiple sites by cAMP-dependent protein kinase A, also known as protein kinase A (PKA)[17]. Likewise, PKA regulates ATGL activity although by an indirect mechanism; ATGL activity is regulated through interaction with the co-regulators comparative gene-identification 58 (CGI-58) and G0/G1 switch gene protein 2 (G0S2)[17]. Thus, activation is dependent on complex formation with CGI-58 but in the basal state CGI-58 is sequestered in a complex with PLIN1. When PKA is activated it phosphorylates PLIN1, leading to the release of CGI-58 and subsequent activation of ATGL. Conversely, G0S2 is a negative regulator of ATGL activity, and the inhibitory effect of this protein is overriding activation by CGI-58 [18]. Being regulated primarily at the protein expression level GOS2 is thought to act as a key regulator of overall ATGL capacity, rather than a component of acute lipolytic cascade [17]. It is unknown if these regulatory mechanisms are affected during the acute phase of inflammation and sepsis.

The present study was designed to define, which intracellular mechanisms become activated in adipose tissue concurrent with the increased rate of lipolysis observed in the initial stages of acute inflammation, using bacterial endotoxin in a human model to replicate these metabolic events under controlled conditions.

## Methods

### Subjects

Data originate from a randomized controlled study from which data regarding protein metabolism have been published [19]. The study design included 3 experimental settings: (i) Saline control (*Placebo*), (ii) LPS administration (*LPS*) and (iii) LPS and amino acid administration (*LPS+A*). Only data from study arm (i) and (ii) during non-insulin stimulated conditions are reported here. Eight healthy, young, and lean male subjects were included in this study. They were eligible for inclusion if they were of male gender, were healthy without regular intake of medication, were between 25 and 40 years of age, and had a body mass index (BMI) between 20 to 30 kg•m^-2^. All subjects were screened using a medical interview and a physical examination including electrocardiography and a blood test screen. An informed written and oral consent was obtained for each subject. Both study days were performed under the same conditions in the same laboratory at Aarhus University Hospital, Denmark. Subjects were without febrile illness the preceding week of each study day and did not exercise 48 hours before each visit. Subjects arrived by car at 07:00 AM after an overnight fast.

### Ethics

The Danish Ethical Committee approved the study (1-10-71-410-12) and both written and oral consent was obtained before inclusion of subjects in the trial. The study was conducted in accordance with the principles stated in the Declaration of Helsinki.

### Design

**Fig 1:**
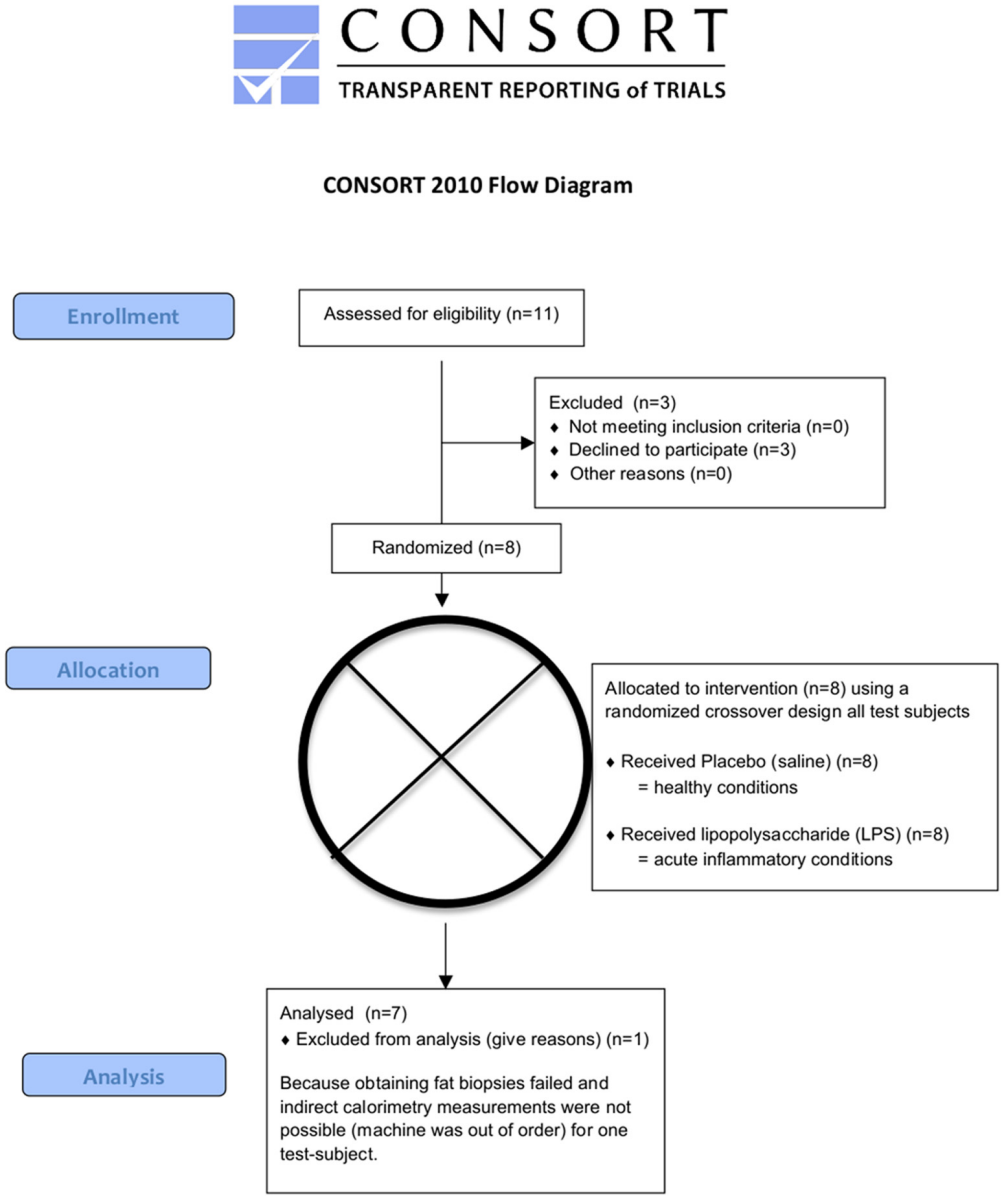
CONSORT flowchart for the trial.

A minimum of 21 days separated the trials for each subject. The primary investigator enrolled all test subjects using a computerized program to randomize interventions. All subjects were blinded in regards to interventions, but could distinguish interventions due to the subjective symptoms following LPS administration. Four of the test subjects received LPS during their first trial followed by Placebo during the second trial and the other four test subjects received Placebo first followed by LPS (Fig 1). During each trial intravenous catheters were inserted in both cubital veins and one in a dorsal hand vein for intravenous infusions and blood sampling.

### Lipopolysaccharide (LPS)

A bolus (1 ng/kg or 10 U/kg) of *E*. *coli* endotoxin (10,000 USP Endotoxin, lot H0K354; The United States Pharmacopeial Convention, Inc., Rockville, Maryland) was given during a two minute period at *time = 0 min* and followed by a 10 ml saline infusion.

### Palmitate tracer and serum concentration of FFA

**Fig 2:**
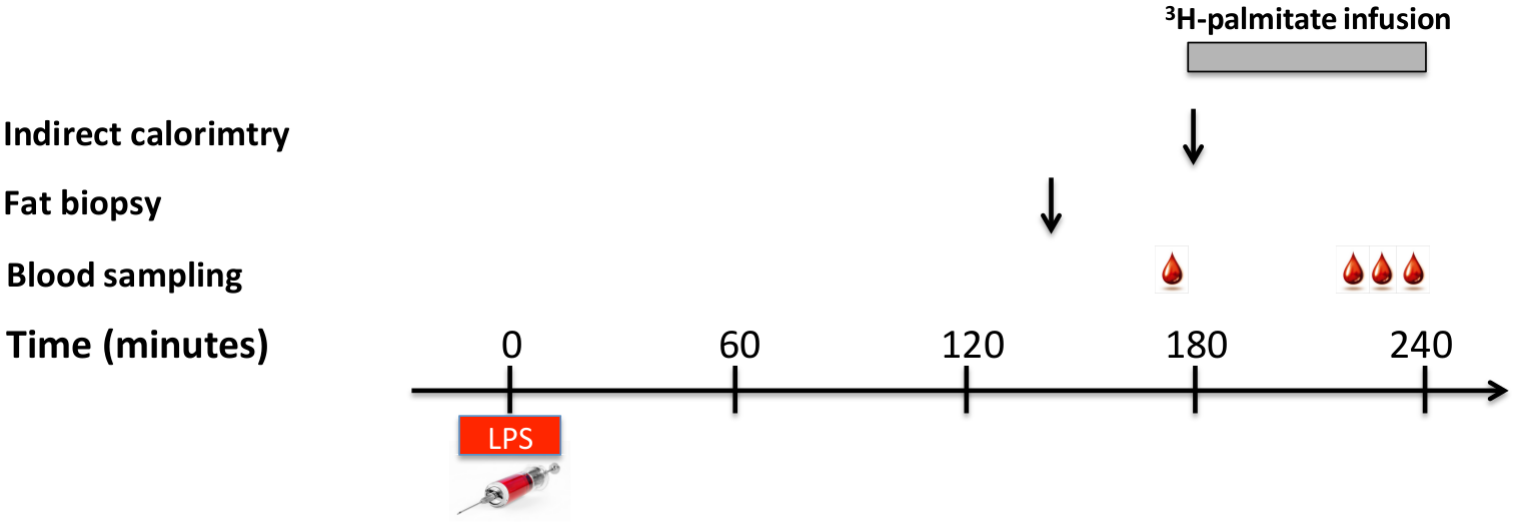
Flowchart showing the time course for the trial days. At *time = 0 min* a bolus of isotonic saline (Placebo) or a bolus lipopolysaccharide (LPS) was given.

A continuous infusion of ^3^H-palmitate (infusion rate 0.3 μCi/min) was given at *time = 180–240 min* (Fig 2). Whole body palmitate calculations were based on Steele’s non-steady state equation [20]:

$$Ra_{\text{palmitate}}=\;\frac{i_{palmitate}}{SA}\;-\;\left(\frac{Vd*c}{SA}*\frac{\Delta SA}{\Delta t}\right)$$

Where *Ra*_palmitate_ is the rate of appearance for palmitate, *i*_*palmitate*_ is the infusion rate of palmitate tracer, *SA* is the specific activity of ^3^H-palmitate, *Vd* is the total distribution volume, *c* is the total concentration of palmitate, *ΔSA* is the difference in specific activity of ^3^H-palmitate between samples, and *Δt* is the time between these samples. *Vd* was estimated assuming that the plasma volume constitutes 5% of total body weight and multiplied with 1.8 as shown by others [21]. Blood samples were collected before starting the ^3^H-palmitate infusion and again at *time = 220*, *230*, *and 240 min*.

The mean serum concentration of FFA were quantified using blood collected at *time = 220*, *230*, *and 240 min* using the *in vitro* enzymatic colorimetric method assay NEFA-HR(2) in accordance with manufactures guidelines (Wako Chemicals GmbH, Germany).

### Indirect calorimetry

An Oxycon Pro calorimeter (Intramedic) with a canopy was applied at *time = 180 min* (Fig 2). A 15-minute period collection of respiratory gases was used to estimate resting energy expenditure (EE) and the respiratory quotient (RQ). Fat oxidation rates were calculated as previously described [22]. Due to technical problems, calorimetry measurements were only completed in 7 subjects.

### Tissue biopsy and western blot analysis

An abdominal subcutaneous adipose tissue biopsy was obtained at *time = 135 min*. The biopsy was immediately washed with isotonic saline to avoid contamination of blood and snap-frozen in liquid nitrogen before storage at -80°C. Fat biopsies were successfully obtained and blotted for n = 7.

All samples were homogenized in a buffer with a 7.4 pH-level and containing 50 mM HEPES, 137 mM NaCl, 10 mM Na_4_P_2_O_7_, 20 mM NaF, 5 mM EDTA, 1 mM MgCl_2_, 1 mM CaCl_2_, 2 mM NaOV, 5 mM NAM, 10 μM TSA, 1% HALT, 1% NP-40, and 10% glycerol. Samples were centrifuged at 14,000 *g* for 20 minutes.

Western blot analyses were used to measure relative contents and phosphorylation of relevant protein targets using the BioRad Criterion system (BioRad). Primary antibodies used were ATGL (GTX62840, GeneTex), CGI-58 (ab183739, Abcam), phosphorylated HSL Ser^552^, Ser^554^, and Ser^650^ (4139, 4137, and 4126 Cell signaling), HSL (4107 Cell signaling), Phospho-PKA Substrate (9624 Cell signaling), PLIN1 (PA1-1052 ABR), G0S2 (sc-133424 Santa cruz), phosphorylated Akt at Thr^308^ and Ser^473^ (9275 and 9271 Cell signaling), Akt (4691, Cell signaling), phosphorylated AS160 at Thr^642^ (4288, Cell signaling), AS160 (07–741, Upstate), and PTEN (9188, Cell signaling). The same membrane was exposed to both Phospho-PKA Substrate antibody and PLIN1 antibody to identify the band representing PKA-phosphorylation of PLIN1. This method has been validated by others using a PLIN1 knock-out model [23].

Control for equal loading was performed using the stain-free technology [24]. Proteins were visualized and quantified using Image Lab 5.0, Bio-Rad laboratories. Data are presented as dot plots showing each subject’s quantification as a ratio compared to the median value for the Placebo group.

### RNA extraction and qPCR for mRNA analysis

RNA was extracted using TRIzol (Gibco BRL, Life Technologies, Roskilde, Denmark) and homogenized with one tungsten bead (Qiagen) using a Mixer Mill. A NanoDrop 8000 Spectrophotometer (Thermo Scientific Pierce, Waltham, Maine, USA) was used to quantify RNA by measuring absorbance at 260 and 280 nm with a ratio ≥1.9. Integrity of the RNA was checked by visual inspection of the two ribosomal RNAs on an agarose gel. A Verso cDNA kit (cat# Ab-1453, Thermo Fischer Scientific) with random hexamer primers was used to synthesize the cDNA. Duplicate PCR-reactions were performed using a KAPA SYBR^®^ FAST qPCR Kit (Kapa Biosystems, Inc. Woburn, MA, USA) in a LightCycler 480 (Roche Applied Science) using the following protocol: One step at 95°C for 3 min., then 95°C for 10 sec., 60°C for 20 sec. and 72°C for 10 sec and finally a melting curve analysis was performed. The increase in fluorescence was measured in real time during the extension step. The relative gene expression was estimated using the default “Advanced Relative Quantification” mode of the software version LCS 480 1.5.1.62 (Roche Applied Science) and specificity of the amplification was checked by a melting temperature analysis.

The following primer pairs were designed using QuantPrime [25]: ATGL: ACCTCAATGAACTTGGCACC and CAACGCCACGCACATCTA length = 122bp, G0S2: CGAGAG-CCCAGAGCC GAGATG and AGCACCACGCCGAAGAG length = 137bp, CGI58: TGTCAGCCG-GCTTCGAGATAAG and ACCAGTTAGCCATCCTGACCTCTC length = 113 bp.

The housekeeping gene, beta2microglobulin, was amplified using this primer pair: b2MG: GAGGCT-ATCCAGCGTACTCC and AATGTCGGATGGATGAAACCC, length = 111bp. Expression level of this housekeeping gene was similar between the Placebo and LPS. All primers were from DNA Technology (Risskov, Denmark). A similar set-up was used for negative controls, except that the reverse transcriptase was omitted and no PCR products were detected under these conditions. Adipose tissue for PCR analysis was only performed in 6 subjects due to lack of adipose tissue.

### Statistics

Data are presented as means or medians with a 95% confidential interval (CI-95%) or range when appropriate. All statistical analyses and graphs were made using Stata 13 (College Station, Texas, USA) and SigmaPlot 11 (San Jose, California, USA). A paired two-tailed students t-test was used to compare the two study arms. Normal distribution was ensured by examination of QQ-plots. Data were logarithmic converted if QQ-plots indicated unequal distribution. If data still were not normally distributed a Wilcoxon signed-ranked test was used. P-values < 0.05 were considered significant.

## Results

### Subjects

The test subjects had a median age of 26 years (range: 25–32), a median BMI of 23 kg•m^-2^ (range: 22–26), and a median body weight of 79 kg (range 68–85 kg) as reported previously [19]. LPS administration caused a significant increase in heart rate, temperature, and serum concentrations of tumor necrotic factor (TNF)-α, interleukin (IL)-6, IL-10, cortisol, GH, glucagon, FFA, and lactate compared with Placebo as reported previously [19]. Furthermore, serum insulin concentrations 15 min prior to fat biopsy sampling were 14.9 pmol/l during LPS and 31.0 pmol/l during Placebo (p<0.05).

### Lipolysis and lipid oxidation

**Fig 3:**
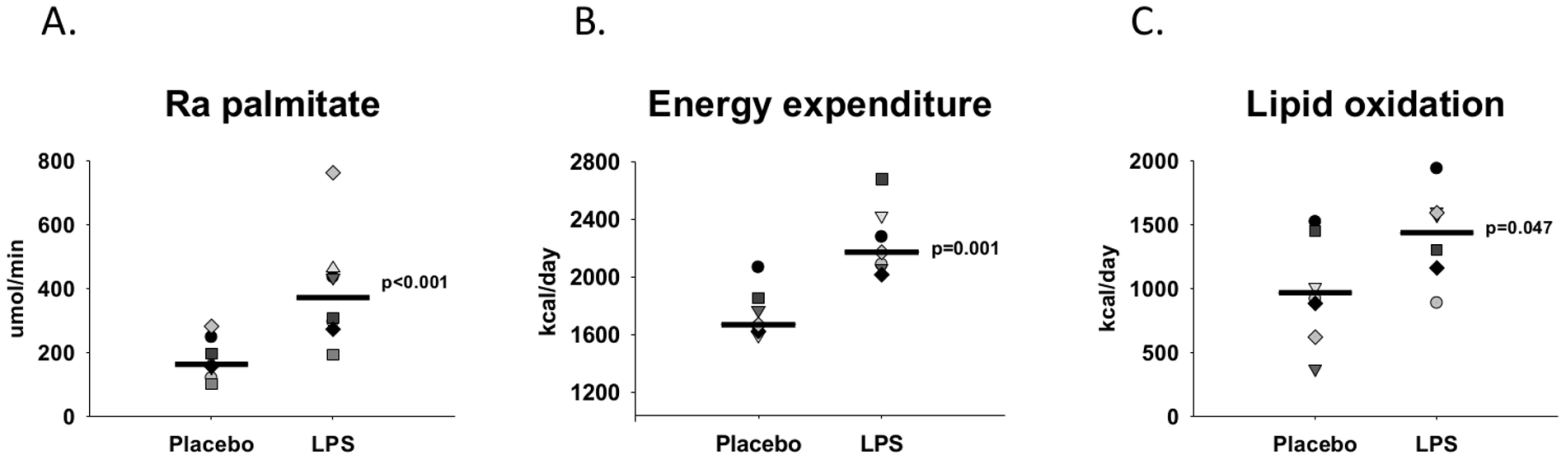
Metabolic measures. Data are shown as dot-plots for each subject during the control conditions with saline administration (Placebo) and the day with lipopolysaccharide administration (LPS). A black horizontal bar indicates the median value for each group. A**.** Ra_palmitate_ (n = 8), **B.** lipid oxidation rates from indirect calorimetry measurements (n = 7), and **C.** energy expenditure from indirect calorimtry measurements (n = 7). Paired sample t-*tests* were used to compare groups. *Ra*_*palmitate*_ *= rate of appearance of palmitate*, *FFA = free fatty acids*.

Absolute serum concentrations of FFA have been reported previously [19] and showed that LPS administration caused a mean increase of 90% (CI-95%: 37–142, p = 0.005) compared with placebo. *Ra*_palmitate_ showed a median increase of 117% (CI-95%: 77–166, p<0.001) during LPS compared with Placebo (Fig 3). In addition, LPS caused a mean increase in the lipid oxidation rate of 49% (CI-95%: 1–96, p = 0.047) and a median increase in energy expenditure of 28% (CI-95%: 16–42, p = 0.001) compared with Placebo.

### Adipose tissue signaling

**Fig 4:**
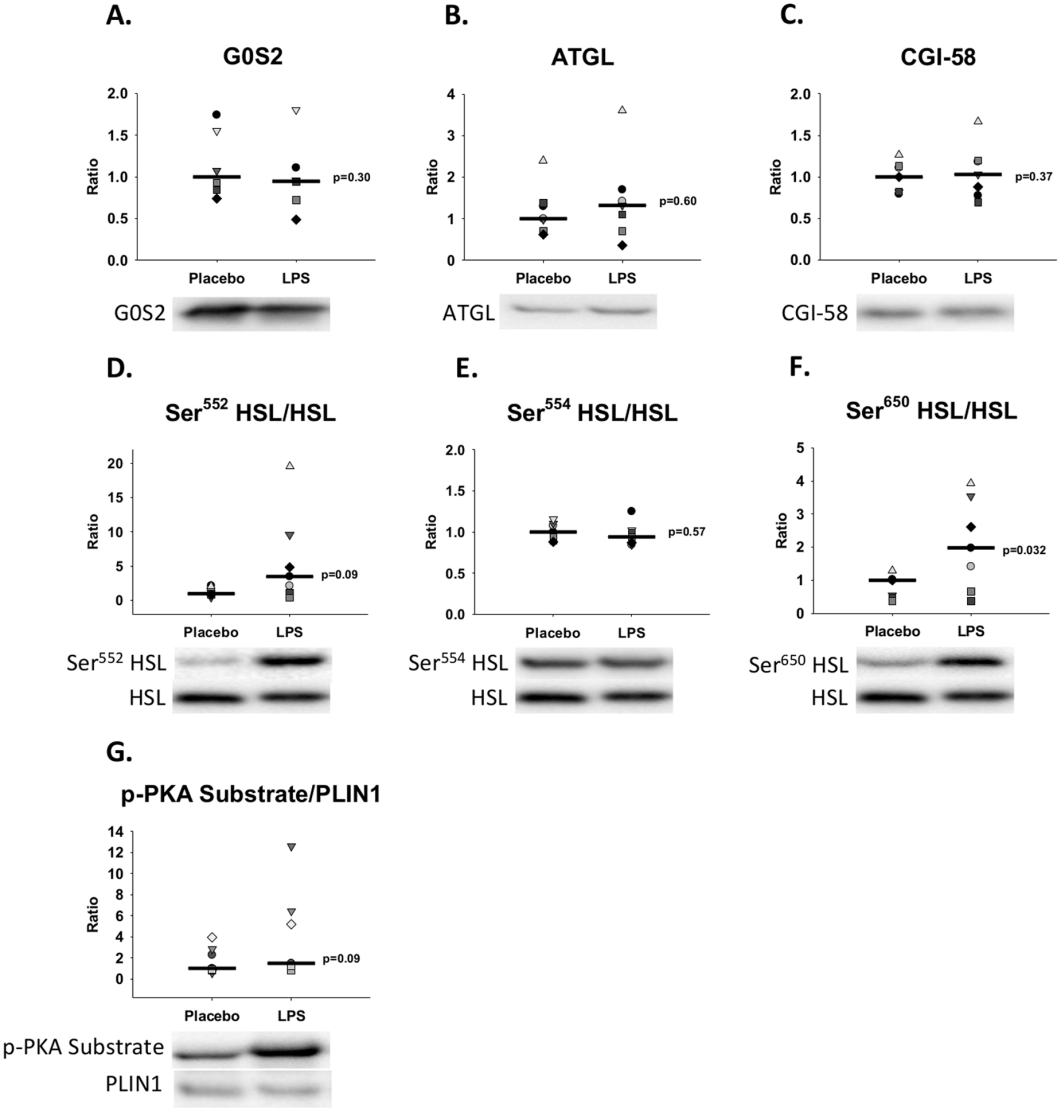
Western blot analyses of subcutaneous abdominal fat tissue biopsies. Representative western blots in abdominal adipose tissue during control conditions (Placebo) and during lipopolysaccharide (LPS) induced endotoxemia (n = 7). Data are presented as the ratio change compared to the median value for the Placebo condition. The black horizontal bars indicate the median value for each group (= 1 for Placebo in all graphs). Paired sample t-test was used to compare groups. **A.** *G0S2 = G0/G1 switch protein 2*, **B.** *ATGL = adipose triglyceride lipase*, **C.** *CGI-58 = comparative gene identification-58*, **D.,E.,** and **F.** *HSL = hormone sensitive lipase*, *and* **G.** *p-PKA Substrate = Phospho-PKA (protein kinase A) Substrate*.

**Fig 5:**
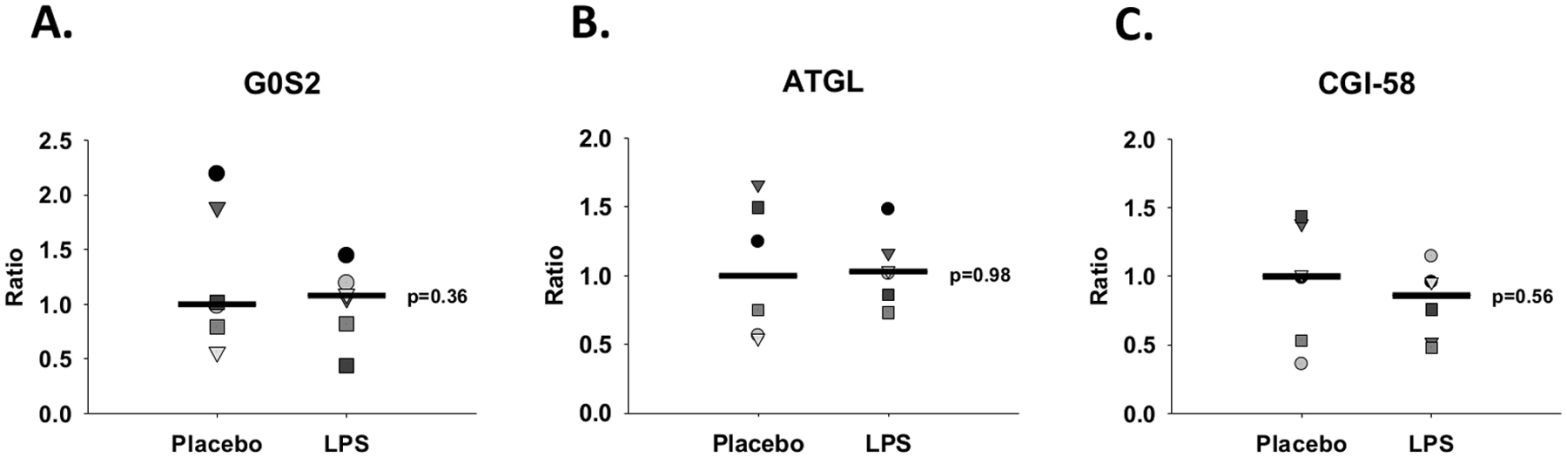
qPCR measurements of subcutaneous abdominal fat biopsies. Quantitative PCR measurements (n = 6) of mRNA are shown for control conditions (Placebo) and during lipopolysaccharide (LPS): **A.** *G0S2 = G0/G1 switch protein 2*, **B.** *ATGL = adipose triglyceride lipase*, *and* **C.** *CGI-58 = comparative gene identification-58*.

Western blot quantification of subcutaneous abdominal fat tissue showed an increase in HSL phosphorylation at Ser^650^ (p = 0.032) during LPS compared with Placebo (Fig 4). In parallel to this finding, HSL phosphorylation at Ser^552^ (p = 0.09) and PKA phosphorylation of PLIN1 (p = 0.09) also tended to increase during LPS compared with Placebo. We did not detect changes between groups in regards to protein levels of ATGL, CGI-58, and G0S2 as well as HSL phosphorylation at Ser^554^ (p>0.05). In agreement with these findings, quantitative PCR analysis showed no difference of ATGL, G0S2, and CGI-58 expression between groups **(**Fig 5**,** p>0.05).

**Fig 6:**
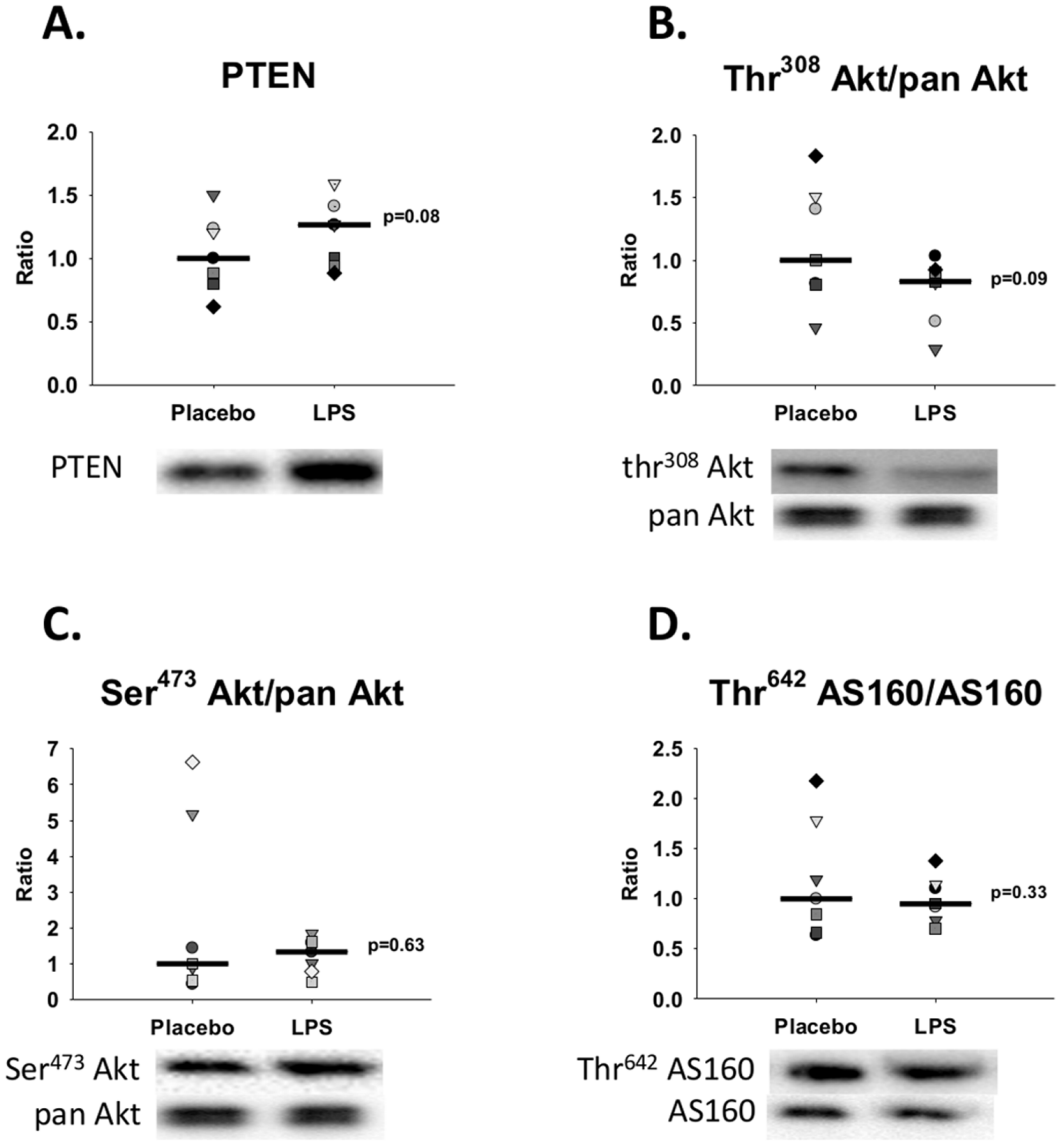
Western blot analyses of subcutaneous abdominal fat tissue biopsies. Representative western blots in abdominal adipose tissue during control conditions (Placebo) and during lipopolysaccharide (LPS) induced endotoxemia (n = 7). Data are presented as the ratio change compared to the median value for the Placebo condition. The black horizontal bars indicate the median value for each group (= 1 for Placebo in all graphs). Paired sample t-test was used to compare groups. **A.** *PTEN =* p*hosphatase and tensin homolog*, **B.** and **C.** *Akt*, *and* **D.** *AS160 = Akt substrate of 160 kDa*.

Phosphatase and tensin homolog (PTEN) tended to increase (p = 0.08) and phosphorylation of Akt at Thr^308^ tended to decrease (p = 0.09) during LPS compared with Placebo. Phosphorylation of Akt (protein kinase B) at Ser^473^ together with phosphorylation of AS160 at Thr^642^ did not differ between groups (Fig 6).

Protein expression of PLIN1, HSL, panAkt, AS160, and β-actin did not differ significantly between groups (p>0.05, data not shown).

## Discussion

In this study, we demonstrate how endotoxin administration causes acute and marked increments in the circulating levels and turnover of FFA concentrations together with increased lipid oxidation and energy expenditure. These changes were associated with increased phosphorylation of HSL at Ser^650^. Additionally, we also detected trends towards increased phosphorylation of HSL at Ser^552^ and increased PKA phosphorylation of PLIN1. These findings may suggest that increased PKA activity is the driving mechanism behind the increased lipolysis seen in the early stages of acute inflammation and sepsis but are still speculative and needs to be confirmed by other human clinical randomized trials.

Our findings are in accordance with earlier studies showing increased lipolysis in the early phase of sepsis and endotoxemia [6–8]. Even though endotoxemia is known to cause increased lipolysis, the mechanisms responsible for this response are poorly understood. Despite the complex interaction between the numerous pro-lipolytic hormones and cytokines, they all eventually stimulate PKA-mediated phosphorylation of PLIN1 and HSL [10,17]. A study by Anthonsen *et al* [26] documented rat HSL phosphorylation at Ser^563^ and Ser^660^ (Ser^552^ and Ser^650^ in human HSL) in cells stimulated with isoproterenol and later it was shown that phosphorylation of PLIN1 by PKA is a decisive step in the activation of lipolysis [27].

A bolus administration of LPS is known to cause an acute increase in epinephrine, GH, and cortisol [28,29], as also observed in our study [19]; all of these hormones are pro-lipolytic. In addition, a study in humans employing direct infusion of LPS into the femoral artery has shown that LPS *per se* increases lipolysis, as judged by increased net palmitate release from the infused leg [11]. Insulin is known to be the main inhibitor of lipolysis [27], and serum insulin concentrations were elevated nearly two-fold during Placebo compared with LPS in our study [19] thereby also favoring increased lipolysis during LPS. These hormonal signals may be further amplified by the borderline increase of PTEN (a known inhibitor of insulin signaling) in our western blot results and the borderline decrease of phosphorylation of Akt (a known downstream effector in the insulin signaling cascade) at Thr^308^ during LPS compared with Placebo. This could indicate that both increased pro- and decreased anti-lipolytic agents may contribute to the increased lipolysis during the acute phase of inflammation. This is primarily a speculative consideration as results per definition were not significantly different between groups (p>0.05), which may be due to the lack of statistical power often limiting human endotoxin trials.

We used both quantitative PCR and western blot to measure G0S2, ATGL, and CGI-58 without finding any differences between LPS and Placebo. Considering the short time-interval between LPS administration and fat biopsy sampling (*time = 135 min*), it seems plausible and predictable that we only found differences in posttranslational protein modifications (e.g. phosphorylations) and no differences or trends when quantifying protein levels. We have previously shown that ATGL and G0S2 expression are regulated during prolonged fasting (72 h) but not affected by an acute exercise bout [30]. Moreover, in cultured adipocytes G0S2 expression is decreased substantially by exposure to TNF-α for 8 hours [18]. These observations further support the hypothesis that remodeling of the lipolytic cascade at the level of protein expression requires persistent, long-term stimulation of lipolysis. It remains intriguing, why lipolysis is increased during the first 24 h of sepsis but then decreases to lower than normal rates after four days of admission [6]. Future studies investigating this issue are necessary to fully understand how lipid mobilization is regulated during the time-course of an infection.

Our borderline significant results (0.05<p-value<0.10) in all likelihood are due to low statistical power. The number of participants in studies using LPS administration is limited by ethical considerations. In addition, the fat biopsies time-points were performed relatively early (fig 1, time = 135) because we aimed at identifying the initial adipocyte signaling modifications responsible for increasing the rate of lipolysis; and it has been shown by others that the inflammatory effects of LPS peaks 60–120 minutes following exposure [31]. It is evidently possible that we would have observed a more profound intracellular adipocyte signaling response and changes in protein expression levels if the biopsies had been performed later.

We used lean healthy men as test subjects, which may imply that our results cannot necessarily be extrapolated to other experimental or clinical situations. Furthermore, these findings were made using subcutaneous abdominal fat biopsies and we therefore do not know if our findings also apply to regulation in other adipose tissue depots. Experiments in rats suggest that visceral adipose tissue is less responsive to endotoxemia than subcutaneous adipose tissue [32] while human studies have found that visceral adipose tissue is more responsive to epinephrine and less responsive to insulin than subcutaneous adipose tissue [33,34].

In conclusion, we found that LPS administration causes an acute increase in lipolysis, FFA concentrations, energy expenditure, and lipid oxidation rates, which is associated with adipose tissue stimulation of pHSL at ser^650^. On a more speculative note it seems as if the initial triggering mechanisms of adipose tissue lipolysis in the acute phase of inflammation and sepsis seem to proceed through PKA-dependent activation of the classical lipolytic cascade.

## Supporting Information

S1 File Original study protocol in English. (DOCX)

S2 File Original study protocol in Danish. (DOCX)

S3 File CONSORT checklist. (DOC)

## Abbreviations

Akt: protein kinase B
ATGL: adipose triglyceride lipase
BMI: body mass index
CGI-58: comparative gene identification-58
EE: energy expenditure
FFA: free fatty acids
G0S2: G0/G1 switch protein 2
GH: growth hormone
HSL: hormone senative lipse
IL: interleukin
Ra_palmitate_: palmitate rate of appearance
RQ: respiratory quotient
PKA: cAMP-dependent protein kinase A
PLIN1: perilipin 1
PTEN: phosphatase and tensin homolog
TAG: triacylglycerol
TNF- α: tumor necrotic factor alpha
