# Supplementary material for: Regulation of Lipolysis and Adipose Tissue Signaling during Acute Endotoxin-Induced Inflammation: A Human Randomized Crossover Trial
Source: PLoS One. 2016 Sep 14;11(9):e0162167. doi: 10.1371/journal.pone.0162167 (PMC5023116; doi:10.1371/journal.pone.0162167)
Supplement: S1 File — (DOCX) [file pone.0162167.s001.docx]

**Keto-metabolites to maintain protein and muscle mass during acute illness in humans - leucine, hydroxymethylbutyrat (HMB) and 3-hydroxybutyrat (3-OHB) as anabolic nutrients.**

**Place:**

Medical science laboratory

Medical department MEA (Endocrinology and Diabetes)

Århus Hospital NBG, Århus University hospital

**PROJEKTDELTAGERE:**

MD, Ph.d.-student Nikolaj Fibiger Rittig (investigator)^1^

MD, Ph.d.-student Ermina Bosnjak^1^

MD, Ph.d. Niels Jessen^1,2^

Professor, Dr. Med. Jens O. Jørgensen^1^

Professor, Dr. Med. Niels Møller ^1^ (main supervisor)

1. Medical department MEA (Endocrinology and Diabetes), Århus University Hospital

2. Clinical Pharmacology, Aarhus University

**BAGGROUND:**
On the 23´rd of august 2011 it was stated in the New England Journal of Medicine that nutritional clinical trials concerning critical ill is warranted^1,2^. Critical illness is catabolic with an increased muscle breakdown^3,4^. The outcome in regards to death, sickness and recovery from acute critical illness is depended on the degree of muscle catabolism and protein loss^3,4^. It is unknown how ketone bodies and amino acids affects patients during acute critical illness. The amino acid leucine is metabolised to the ketones hydroxymethylbutyrate (HMB) and 3-hydroxybutyrate (3-OHB). These substrates are shown to be anabolic during different eksperimental models^3,5^.

The loss of protein or muscle depends on the balance between protein synthesis and breakdown, which can be investigated using in vivo tracer techniques. The increased breakdown is often caused by immobilisation and systemic inflammation (increased levels of pro-inflammatory markers such as interleukins and TNF-alpha)^3,6,7^. This combination causes insulin resistance and hyperglycaemia due to higher levels of stresshormones (cortisol and glucagon)^6^. It has been shown that the degree of insulin resistance can be prognostic in a population undergoing surgery^6^, and that it most likely is depended on the degree of muscle breakdown.

The effect of ketones given as a nutritional supplement during acute critical illness is poorly investigated, and therefore it remains unknown which local and systemic effects it might cause.

**Hypothesis:**

We hypothesise that that specific keto-metabollites (Leucine/HMB/3-OHB) affects intracellular signaling pathways that might counteract the progressive protein and muscle loss. This could have prognostic and socioeconomic value. As a “model” for acute illness we will use E. Coli endotoxin to create a systemic response that is comperable with the one seen during acute ilness.

**Aim:**

The primary aim of this study is to investigate the effects of leucine, HMB and 3-OHB during endotoxin induced acute illness. The keto-metabolites will be given as an enriched nutritional supplement developed by ARLA. These substrates have been shown to be anabolic as they increase protein levels, muscle mass and the function of the heart^3,11^. Acute illness is catabolic and protein breakdown has prognostic value^1-4^. The effect of these ketometabolites during acute illness is unknown. If we show improved effects it might have both prognostic and socioeconomic consequences which could open a new global market of nutritional supplements.

**Inclusion criteria:**

- male
- 20 < BMI < 30
- age >25 år
- Written consent before the investigation starts

**Exclusion criteria:**

- Participation in trials using radiation the past year
- Radiation from larger x-ray investigations the past year
- If participants have been immobilised for longer time a doctor/physical therapist must declare the muscles retrained. The participants statement is not enough.
- Allergy for egg or soya oil
- Diabetes
- Epilepsy
- On-going infection
- Immune deficiency
- Heart disease
- Hypertension

Participants will be excluded if:

1. They do not want to participate
2. If they experience serious or intolerable side effects from the interventions or other things during the trial. If they experience serious or intolerable side-effects, the trial for this participant will be stopped and the investigator will arrange relevant follow-up in regards to admission and treatment regime.

Data from excluded participants will be used if possible – it will depend on the time of exclusion. The data that can be analysed will then be used. Excluded participants will be replaced by a new if possible.

**Physical examination**

The screening includes control for inclusion criteria, a physical examination including height, weight, blood pressure and pulse. Furthermore blood sample analysis for haemoglobin, thrombocytes, INR, APTT, CRP, leucocytes, liver enzymes, kidney markers, and a fasting glucose level. This screen will be performed at least one week before participating in the trial.

**Study design:**

The study design is a randomised, open/single blinded in regards to amino acid infusion. Participants and lab personal who makes biochemical analysis on blood samples and tissues are blinded.

**Methods:**

Eight healthy participants will be included, and investigated 5 times each in random order. Each trial day starts with an overnight fast and starts in the morning. Participants are instructed to avoid physical activities 48 hours before the trial and to arrive in Taxa. After the trial participants will be monitored for at least one hour.

**Interventions:**

- Placebo (saline)
- Endotoxin, US standard reference E.Coli (Bolusinfusion af 0.2 ng/kg twice) + Placebo p.o.
- Endotoxin, US standard reference E.Coli (Bolusinfusion af 0.2 ng/kg twice) + Amino acids p.o.
- Endotoxin, US standard reference E.Coli (Bolusinfusion af 0.2 ng/kg twice) + Valle /Leucine p.o.
- Endotoxin, US standard reference E.Coli (Bolusinfusion af 0.2 ng/kg twice) + HMB p.o.

The chosen endotoxin dose is discussed in “ethic considerations”

***This section is not part of the original protocol, but for your information:***

***“Two supplemental protocols were send to the ethics committee and were accepted before the trial started: The first was made to change the dose of LPS to a single bolus of 1 ng/kg, and the second to change the design to only involve 3 interventions as it came to our attention that LPS causes decreased gastric emptying, nausea and vomiting. For these reasons it was decided that the nutritional supplement should be given intravenously and only contain amino acids. The placebo day was an iv. saline infusion.”***

**Hyperinsulinemic euglykaemic clamp (HEC):**

During the hyperinsulinemic euglycaemic clamp an infusion of supra-physiological insulin (1.0 mU/kg/min) will be started. To clamp the blood glucose level at 5 mmol/l an infusion of 20% glucose will be started in variable rates. Blood glucose levels will be measured every 10 minutes. The glucose infusion rate (M-value) is depended on the insulin sensitivity

**Trial day:**

t= 0 min.: Intravenous catheters are placed for infusion of interventions, insulin, glucose, tracers and blood sampling. A urine sample is collected. Basal blood samples are taken.

t= 0 – 360 min.: A primed bolus and continuously infusion of ^15^N-phenylalanin, ^3^H_3_-glucose, ^15^N –Tyrosin, ^2^H_4_–Tyrosin (only bolus), and ^2^H_4_- Carbamid tracer (only 240 min.) are started. Interventions are started.

t= 120 min.: Muscle and fat biopsies are obtained from the vastus lateralis and abdomen respectively. A 60 min. intravenously infusion of ^3^H-Palmitat tracer is started.

t=140-160 min: Calorimetry in 20 min.

t= 240 min.: HEC is started.

t=270 min.: Muscle and fat biopsies are obtained.

t= 300 min.: A 60 min. intravenously infusion of ^3^H-Palmitat tracer is started.

t=340-360 min: Calorimetry for 20 min.

t= 360 min.: Urine sample is collected. Blood samples are obtained. Investigation is finished.

**Flowchart;**

**Endpoints:**

Primarily:

- Glucose, amino acids, and lipids are quantified using the arterio-venous tracer method.

- Signalling activation is measured in muscle and fat tissue

Secondary:

- Cytokine levels in plasma

**Blood samples:**

Overall approximately 400 ml of blood will be drawn per trial day (2000 ml overall during the 5 trial days), blood samples will be properly handled and stored at -80º C. Analysis will be made when all eight participants has completed all trial days. A Biobank will be used to storage tissue and blood samples.

**Metabolic analysis:**

Blood sample analysis include Na^+^, K^+^, creatinin, insulin, glucagon, catecholamine’s, cortisol, IGF1, growth hormone, free fatty acids*, glycerol*, glucose*, urea, amino acid tracers*, palmitate tracer*. (*=A-V differences).

**Fat metabolic analysis:**

We use a palmitate isotope as tracer. By estimating differences in specific activity of palmitate (the relationship between palmitate-isotope and total palmitate) we get an estimate of palmitate metabolism. We use a continuously i.v. infusion of the isotope [9,10^3^H]-palmitate 0,3 μCi/min for 2 x 60 minutes.

**Amino acid analysis:**

Urine samples and blood samples are collected to estimate urea (as a measure for protein breakdown).

Protein breakdown and synthesis are estimated using the infusion of ^15^N-phenylalanin, ^15^N –Tyrosin og ^2^H_4_–Tyrosin.

*Amino acid kinetics.* We apply previously used (by our department) calculations to estimate amino acid kinetics:

Qflux = *i*[(Ei/Ep) – 1]

Where *i* is tracer infusion rate (mmol kg^–1^ h^–1^), and Ei and Ep are isotope enrichments of the infused tracer and in. For an example, phenylalanine balances are calculated as:

PheBal = (PheA – PheV) * F

Where PheA and PheV are phenylalanine concentrations in the arteries and veins, and F is blood flow.

Regional phenylalanine fluxes are calculated as:

Ra Phe = PheA [(PheEA/PheEV) – 1] * F

Where PheEA and PheEV are phenylalanine isotope enrichments in arteries and vein.

Rd is defined as: Rd Phe = Phe Bal + Ra Phe.

**Indirect calorimetry**

Using a Deltatrac monitor we measure participants oxygen consumptions and carbon dioxide production. These values are used to calculate the respiratory quotient (RQ). This measure is used to estimate glucose and fat oxidation. The method is non-invasive and only requires that the participant lies in bed with a canopy over his head.

**Immunologic analysis:**

Blood samples will be analysed using a Luminex® kit for cytokines: IL-1, IL-2, IL-4, IL-6, IL-8, IL-10, IL-12, IFN-γ, TNF-α.

Venous blood is drawn for phenotype characterisation, adhesion molecules, and intracelleular cytokine production between the circulating lymphocytes populations using a multicolour flow cytometry.

**Muscle biopsy:**

Two muscle biopsies will be obtained per trial. The lateral area of the upper leg is disinfected and local anaesthesia is applied (lidokain 10 mg/ml) to the fascia, subcutes and skin. A scalpel is used to make a small incision through skin and fascia. A Bergström cannula is used to obtain approximately 300 mg muscle tissue from the m. vastus lateralis. The muscle biopsy is immediately frozen.

Signalling pathways on muscle biopsies include insulin signalling (PI3 kinase, Akt and more), amino acid signalling SOCS/STAT systems: activated STAT5, MAP-kinases activity, SOCS mRNA, IGF-I mRNA, PI3-kinase activity, AS160 phosphorylation, NF-κB activity, and mRNA quantification and more.

**HÅNDTERING OG ARKIVERING AF DATA:**

Data will be kept anonymous. Data will be stored at department MEA, Aarhus University Hospital, NBG. Data will be kept for 15 years from the trial ends.

**TRIAL RISKS:**

Every needle injection is with a minor risk of infection. The muscle biopsies are associated with local pain/soreness that will decline during the following couple of days. If signs of skin infection appear (redness, warmth, soreness and swelling) or even doubt about this, the participant must contact the primary investigator MD Nikolaj Fibiger Rittig (tlf +45 61714731) or the doctor on call at department MEA (call Aarhus University Hospital at +45 7845000 and ask to speak with the doctor on call).

Of the hundreds of muscle samples performed at our department we have only experienced minor side-effects. We have only experienced one episode with loss of muscle mass from the area the sample was taken. This was a temporary complication and did not affect standing, walking or exercise/sports.

Endotoxin originates from certain bacteria’s cell wall and is widely used to create a controlled degree of inflammation in healthy participants. This serves as an opportunity to investigate different mechanisms during the acute phase of inflammation/sickness. A larger dose of endotoxin gives flue-like symptoms: Discomfort, chills, muscle soreness, fever, and faster breathing. The dose given in our trial is expected to give a mild degree of these symptoms.

There is a minor risk for hypoglycaemia in the following hours and therefore a meal is served after the trial is completed. Symptoms such as palpitations, sweating, discomfort, and tremor are seen as signs of hypoglycaemia. Blood glucose levels are measured the following hour of the trial and glucose is infused until glucose levels are stable. Sugar tablets are given as a precaution to hypoglycaemia events after the observations period.

We use small amounts of radioactive labelled fat and sugar to investigate the metabolism. The overall radiation during a trial day corresponds 15 % of the annually background radiation. Theoretically this increases the lifetime risk for cancer with only 0.009 %. All the investigations performed are well known in our lab.

There can come unexpected complications during the trials.

**RECRUITING:**

The website [www.forsøgsperson.dk](http://www.forsøgsperson.dk) will be used to recruit possible participants. Advertising on Aarhus University and in local news papers can also be used to recruit participants.

**QUALITY CONTROL:**

The trial will be performed in agreement with the GCP (Good Clinical Practice).

The Danish Health and Medicines Authority have verified endotoxin as a so called “tool” to induce inflammation.

We hereby confirm that the study will be performed as stated in this protocol, in accordance with the governmental requirements and laws, in agreement with the GCP. The Regional Ethics Comitee and the Danish Health and Medicine Authority must approve the protocol. The trial will be reported to the Danish Data Council.

The trial will be performed in accordance with Helsinki declaration.

The investigator allows ethic committees or other equivalent authority access to monitor and verify all relevant data from the trial. The trial needs to be approved at the Regional Ethics Comitee, The Danish Health and Medicines Authority, and the Danish Data council.

**ETHICAL CONCIDERATIONS:**

**Ethical considerations in regards to interventions:**

Sepsis, insulin resistance, and protein/muscle loss are central problems during acute critical illness. It remains unknown how these metabolic parameters are affected during the acute phase of inflammation. For this reason, we find that the risks during the trial day is compensated by the possible knowledge we will gain from the trials.

Endotoxin can give inflammatory side-effects such as flue-like symptoms, nausea, fever, headache, muscle soreness^8^, and under extreme situations sepsis and septic chock^9,10^.

We have experience in regards to endotoxin in our lab, and have given it locally in a leg and as a systemic infusion to healthy and sick volunteers. The doses are based on other studies using the same kind of endotoxin^11^.

We do not expect endotoxin to give other symptoms than the flue-like symptoms described and rise in temperature. There has not been reported any serious adverse effects of endotoxin and vital parameters will be monitored and measured during the trial.

**Ethical considerations in regards to methods:**

The overall radiation is approximately 1.0 mSv (per trial), which corresponds to about 15 % of the normal annually background radiation. This corresponds to a theoretically increased lifetime risk of developing cancer of 0.009%. For the average Dane this increase is from 25.00% to 25.009% for every trial day.

The muscle biopsies are routinely performed at our lab and we have only experienced one case of reversible atrophy.

**SAFETY:**

Before the trial starts participants will be screened.

During the trial a MD and/or a medical laboratory technician will be present to ensure the participants well being and measure vital parameters such as blood pressure, heart rate, temperature, blood glucose, and awareness.

Serious events is defined as: Every medical case regardless dose: Results in death, is life-threatening, results in admission to the hospital or prolonged admission to the hospital, or side effects that results in pronounced abnormalities or disabilities, and in the case of congenital abnormalities.

These serious adverse effects will be reported using the proper formula for clinical trials side effects. Other minor side effects is noted in the protocol for each participant. The investigator will notify the Regional Ethic Committee if we experience any serious events and the time for registration ends when the observation ends as the endotoxin and its side-effects are gone from this moment

Complications will be registered and described using medical objective observations and treated at our science laboratory on a highly specialised hospital. In the case of a serious event, the trial will stop immediately for this participant and he will be properly instructed about the condition and treatment regime. The participant will be monitored and treated until symptoms are gone or the condition is stabilised. Furthermore it must be considered if the trial for all participants must be stopped.

All side effects and adverse events will be noted in the final rapport.

Participants are insured in correspondence to the Danish legal rights concerning health care and will be compensated in accordance with these rights.

**STATISTICS:**

Comparison will be made using standard statistical methods (t-test or non-parametric tests). Time series and effects of interventions will be estimated using repeated measures analysis of variance (ANOVA). P values under 0.05 are considered significant

Power calculations will not be made for all endpoints, but are based on insulin sensitivity during the *clamp* (“M-vallue”).
Number n = 2 x f (α,β) x (σ/ δ)^2^
α is the risk of type 1 errors, β is the risk of type 2 errors, σ is the SD and δ the difference we can meassure. We have earlier found M-values with a SD of 5% and β of 20 % which equals; f = 7.9. If we want to measure a possible difference in M-value of 7 %, then n = 2 x 7.9 x (5/7)2 = 8.01, N = 8.

All data will be kept anonymous and only the primary investigator will be able to decode this.

**GUIDELINES FOR WRITTEN AND ORAL INFORMATION:**

The written and oral information are given in accordance with the Danish Science Authority’s guidelines for obtaining consent from participants in biomedical clinical trials. After reading about the trial, participants will contact the investigator by phone or email to arrange a meeting where the oral information will be given. They will be informed that they are allowed to bring an acquaintance for this meeting. The Central Ethics Comitee has an flyer with information called “Participants rights in clinical trials” which will be send to the recruiting participant together with the written information about the trial before the meeting. This way the recruiting participant can read the written information and bring questions to the oral information meeting. We will actively recommend that the participants read these information.

The oral information will be given by MD, Nikolaj Rittig. This information will be given in a quiet setting with no other personal in the room. Recruiting participants will have the possibility to ask questions and they have the right not to be informed about their own health situation.

It will be outlined that they participate voluntarily and that they at any given time can retreat from the trial without it having any effect on the present or future doctor-patient relationship. After the oral information meeting, the participant will be offered a minimum of one day to consider participating in the trial. If the participants agree to participate they will be asked to fulfil a written formula “Standard Consent” that is made for clinical trials involving a biobank and storage of biological materials. The screening could be done after the oral information has been given if it is most practical.

**FINANCE:**

The trial and payroll costs are financed from the “keto project”, journal number 11-115840 from the Danish Council for Strategic Research. The main responsible and promoter of the study is Proffesor, Doctor Niels Møller.

Arla Foods group P/S has given financial support to the overall ”Keto projekt” by producing and developing nutritional products for an estimated value of 800.000 kr. There is no conflict of interest between the supporting party or investigators.

.

**COMPENSATION:**

Participants will be compensated with 1200 kr per trial day, so 6000 kr for all 5 trials.

These reimbursements are taxable. Participants will be equipped with a Taxa receipt to ensure absence from physical activity on the morning of the trial.

**PUBLICATIONS:**

These clinical trials will result in at least 4 publications in international peer-reviewed journals with Nikolaj Rittig as the first author and Niels Møller as the senior author. Both positive, negative, and inconclusive results will be published.

**REFFERENCER:**

1. Ziegler TR. Nutrition support in critical illness--bridging the evidence gap. *N Engl J Med.* *2011; 365: 562-4.*
2. Fearon KC. Cancer cachexia and fat-muscle physiology. *N Engl J Med. 2011; 365: 565-7.*
3. [Zudin Puthucheary](http://jp.physoc.org/search?author1=Zudin+Puthucheary&sortspec=date&submit=Submit)[^1^](http://jp.physoc.org/content/588/23/4641.long#target-1), [Hugh Montgomery](http://jp.physoc.org/search?author1=Hugh+Montgomery&sortspec=date&submit=Submit)[^2^](http://jp.physoc.org/content/588/23/4641.long#target-2), [John Moxham](http://jp.physoc.org/search?author1=John+Moxham&sortspec=date&submit=Submit)[^3^](http://jp.physoc.org/content/588/23/4641.long#target-3), [Stephen Harridge](http://jp.physoc.org/search?author1=Stephen+Harridge&sortspec=date&submit=Submit)[^4^](http://jp.physoc.org/content/588/23/4641.long#target-4)

and [Nicholas Hart](http://jp.physoc.org/search?author1=Nicholas+Hart&sortspec=date&submit=Submit)[^5^](http://jp.physoc.org/content/588/23/4641.long#target-5) Structure to function: muscle failure in critically ill patients*.* [*J Physiol.*](javascript:AL_get(this,%20'jour',%20'J%20Physiol.');) *2010 Dec 1; 588(Pt 23):4641-8.*

1. Weitzel LR, Sandoval PA, Mayles WJ, Wischmeyer PE. Performance-enhancing sports supplements: role in critical care. *Crit Care*

*Med. 2009 Oct; 37(10 Suppl):S400-9.*

1. Suryawan A, Jeyapalan AS, Orellana RA et al. Leucine stimulates protein synthesis in skeletal muscle of neonatal pigs by enhancing

mTORC1 activation. *Am J Physiol Endocrinol Metab* *2008; 295: E868-875.*

1. Gustafsson UO, Ljungqvist O. Perioperative nutritional management in digestive tract surgery. *Curr Opin Clin Nutr Metab Care.*

*2011; 14: 504-9.*

1. Andreasen AS, Krabbe KS, Krogh-Madsen R, Taudorf S, Pedersen BK, Møller K. Human endotoxemia as a model of systemic inflammation. *Curr Med Chem. 2008; 15: 1697-705. Review*
2. Vesali RF, Klaude M, Rooyackers O, Wernerman J: Amino acid metabolism in leg muscle after an endotoxin injection in healthy

volunteers. *Am J Physiol, Endocrinol Metab 2004, 288: E360-64.*

1. Tracey K, Beutler B, Lowry S, et al: Shock and tissue injury induced by recombinant human cachectin. *Science 1986, 234: 470-474*
2. Tracey K, Fong Y, Hesse D, et al: Anti-cachectin/TNF antibodies prevent septic shock during lethal bacteremia. *Nature 1987, 330:*

*662-664*

1. Taudorf S, Krabbe KS, Berg RM, Pedersen BK, Moller K: Human models of low-grade inflammation: bolus versus continuous infusion of endotoxin. *Clin Vaccine Immunol 2007, 14(3): 250-2551*
2. Cahill GF Jr. Fuel metabolism in starvation. *Annu Rev Nutr* *2006; 26: 1-22.*
