# Supplementary material for: Regulation of Lipolysis and Adipose Tissue Signaling during Acute Endotoxin-Induced Inflammation: A Human Randomized Crossover Trial
Source: PLoS One. 2016 Sep 14;11(9):e0162167. doi: 10.1371/journal.pone.0162167 (PMC5023116; doi:10.1371/journal.pone.0162167)
Supplement: S2 File — (DOCX) [file pone.0162167.s002.docx]

**Keto-metabolites to maintain protein and muscle mass during acute illness in humans - leucine, hydroxymethylbutyrat (HMB) and 3-hydroxybutyrat (3-OHB) as anabolic nutrients.**

**STED:**

Medicinsk Forskningslaboratorium

Medicinsk afd. MEA (Endokrinologi og Diabetes)

Århus Sygehus NBG, Århus Universitetshospital

**PROJEKTDELTAGERE:**

Læge. Ph.d.-studerende Nikolaj Fibiger Rittig (investigator)^1^

Læge, Ph.d.-studerende Ermina Bosnjak^1^

Læge, Ph.d. Niels Jessen^1,2^

Professor, Overlæge, Dr. Med. Jens O. Jørgensen^1^

Professor, Overlæge, Dr. Med. Niels Møller ^1^ (klinisk ansvarlig)

1. Medicinsk afd. MEA (Endokrinologi og Diabetes), Århus Universitetshospital

2. Klinisk Farmakologi, Aarhus Universitet

**BAGGRUND:**
Det blev i tidsskriftet New England Journal of Medicin fra den 23. august 2011 pointeret, at der er et langt større behov for at undersøge vigtigheden af ernæringsmæssig intervention hos akut kritisk syge mennesker^1,2^. Akut kritisk sygdom udgør en katabolsk tilstand med svært proteintab^3.4^. Hos patienter med akut kritisk sygdom er graden af proteintab og muskelsvaghed med til at bestemme dødelighed, sygelighed og restitution^3,4^. Det vides ikke hvordan ketonstoffer og aminosyrer påvirker patienter med akut kritisk sygdom. I kroppen metaboliseres aminosyren leucin til ketonstofferne hydroxymethylbutyrat (HMB) og 3-hydroxybutyrat (3-OHB). Disse 3 metabolitter er anabolske forbindelser, og de er vist at øge protein- og muskelmasse i eksperimentelle modeller^3,5^.

Tab af protein og muskelmasse skyldes enten nedsat syntese eller øget nedbrydning, og disse processer kan måles *in vivo* med bl.a. aminosyre tracers. Oftest skyldes tabet af muskelmasse en kombination af immobilisering og systemisk inflammation som forårsager høje niveauer af proinflammatoriske cytokiner som TNF-alpha (der kan påvirke appetitten)^3,6,7^. Denne kombination medfører insulinresistens og hyperglykæmi bl.a. på grund af høje niveauer af stresshormoner (fx glucagon og cortisol)^6^. Det er vist at graden af insulinresistens har prognostisk værdi hos kirurgiske patienter^6^, og det skyldes formentligt i høj grad det store proteintab der ses i forbindelse med svær insulinresistens.

Effekten af ketonstoffer givet som beriget ernæringsprodukt i forbindelse med akut svær sygdom er ikke beskrevet, og det vides derfor ikke hvilke lokale og systemiske påvirkninger de medfører.

**Hypoteser:**

Vores hypotese er, at specifikke keto-metabolitter (leucin/HMB/3-OHB) påvirker intracellulære signalveje, således at man under akut sygdom vil se mindre tab af protein- og muskelmasse. Dette vil forbedre prognosen og mindske helbredsmæssige omkostninger for samfundet hos patienter med akut svær sygdom. Som en ”markør” for akut sygdom bruger vi E. Coli endotoxin, da vi mener at dette vil fremkalde et systemisk respons der kan sammenlignes med en generel akut sygdomsreaktion.

**Formål:**

Det primære formål med dette studie er at undersøge de metaboliske effekter af ketometabolitterne leucin, HMB og 3-OHB under endotoxin-induceret akut sygdom. Ketometabolitterne vil blive givet som et beriget ernæringsprodukt udviklet af ARLA. I eksperimentelle studier er det vist at disse stoffer øger proteinmængden, muskelmassen og hjertefunktionen^3,11^. Akut sygdom er en katabol tilstand, hvor tabet af protein og muskelmasse har betydning for prognosen^1-4,^ . Det vides dog ikke hvilken effekt ketometabolitterne har i forbindelse med akut sygdom. Hvis ernæringsproduktet viser gavnlige effekter på protein og muskelmasse vil det kunne forbedre prognosen hos akut syge mennesker, reducere samfundsøkonomiske omkostninger i forbindelse med indlæggelse og åbne op for et nyt globalt marked inden for ernæringsprodukter.

**Inklusions kriterier:**

- Hankøn
- 20 < BMI < 30
- Alder >25 år
- Skriftligt samtykke før undersøgelsesstart

**Eksklusionskriterier:**

- Deltagelse i forsøg der anvender ioniserende stråling i et år forud for inkludering i dette forsøg
- Større røntgen undersøgelser i samme periode
- Såfremt der har været immobiliseringsperiode af en underekstremitet, skal man være erklæret genoptrænet af behandlende læge/fysioterapeut for at kunne deltage. Forsøgspersonens ord for at dette er tilfældet, vil være tilstrækkeligt.
- Allergi for æg eller sojaolie
- Diabetes
- Epilepsi
- Pågående infektion
- Immundefekt
- Hjerte/kar sygdom
- Dysreguleret hypertension

Forsøgspersoner vil udgå af undersøgelsen, såfremt:

1. De selv ønsker det.
2. Hvis der forekommer alvorlige eller intolerable bivirkninger fra interventionen eller andet indgivet under forsøget. Såfremt forsøgsperson udvikler alvorlige eller intolerable bivirkninger, vil forsøget for den enkelte deltager blive stoppet og investigator vil foranledige relevant opfølgning, herunder henvisning til relevant behandling.

Data opnået fra udgået forsøgsperson vil blive anvendt, så vidt det er muligt – det vil afhænge af, hvornår i undersøgelsen en given forsøgsperson måtte udgå. De data, det er muligt at analysere sig frem til, vil blive benyttet. Udgåede forsøgspersoner forsøges erstattet af nye forsøgspersoner.

**Helbredsundersøgelse**

Screeningsbesøget omfatter kontrol af inklusionskriterier, objektiv undersøgelse inkl. måling af højde, vægt, blodtryk og puls. Desuden tages blodprøve med henblik på måling af hæmoglobin, trombocytter, INR, APTT, CRP, leukocytter og differentialtælling, levertal, nyretal og faste blod glukose. Undersøgelserne udføres senest 1 uge før forsøgets start.

**Studiedesign:**

Studie-designet er randomiseret, åbent/enkelt blindet hvad angår indgift af aminosyrer, leucinberiget valle og HMB. Forsøgspersoner og bioanalytikere, som udfører de biokemiske analyser på blodprøver og væv, vil være blindede.

**Metode:**

Der inkluderes 8 raske forsøgspersoner, som hver undersøges 5 gange i randomiseret rækkefølge på 5 forskellige forsøgsdage. Forsøgspersonerne møder fastende om morgenen på forsøgsdagen. De instrueres i ikke at dyrke motion 48 timer før forsøget, og skal ankomme i taxa. Forsøgspersoner vil efter endt forsøg blive observeret på sygehuset i 1 time.

**Interventioner:**

- Placebo (saltvand)
- Endotoxin, US standard reference E.Coli (Bolusinfusion af 0.2 ng/kg af to omgange) + Placebo p.o.
- Endotoxin, US standard reference E.Coli (Bolusinfusion af 0.2 ng/kg af to omgange) + Aminosyrer p.o.
- Endotoxin, US standard reference E.Coli (Bolusinfusion af 0.2 ng/kg af to omgange) + Valle /Leucin p.o.
- Endotoxin, US standard reference E.Coli (Bolusinfusion af 0.2 ng/kg af to omgange) + HMB p.o.

Valget af de ovennævnte endotoxin-doser diskuteres i afsnittet ”Etiske overvejelser” under ”Etiske overvejelser med hensyn til intervention”.

**Hyperinsulinæmisk euglykæmisk clamp (HEC):**

Ved hyperinsulinæmisk euglukæmisk clamp, infunderes insulin i en suprafysiologisk dosis (1,0 mU/kg/min) intravenøst. For at fastholde blodsukker på 5 mmol/l, kontrolleres dette hvert 10 minut, og der infunderes 20 % glukose i. v. Den hastighed hvormed man infunderer kaldes en M-værdi og er direkte korreleret til insulin-følsomheden.

**Undersøgelsesprogram:**

t= 0 min.: Der anlægges tre perifere venflons (med henblik på infusion af interventioner, insulin, glukose og tracere, samt blodprøvetagning)**.** Forsøgspersonen har vandladning. Der tages basalblodprøver.

t= 0 – 360 min.: Der gives mætningsbolus og kontinuerlig intravenøs infusion af ^15^N-phenylalanin, ^3^H_3_-glucose, ^15^N –Tyrosin, ^2^H_4_–Tyrosin (kun bolus) og ^2^H_4_- Carbamid tracer (kun i 240 min.). Herefter indledes 360 minutters systemisk infusion af interventioner.

t= 120 min.: Der tages muskelbiopsi på én underekstremitet og efterfølgende fedt biopsi fra maveskindet (se venligst senere). 60 min. intravenøs infusion af ^3^H-Palmitat tracer.

t=140-160 min: Kalorimetri i 20 min.

t= 240 min.: HEC påbegyndes.

t=270 min.: Der tages muskel biopsi og efterfølgende fedtbiopsi.

t= 300 min.: 60 min. intravenøs infusion af ^3^H-Palmitat tracer.

t=340-360 min: Kalorimetri i 20 min.

t= 360 min.: Forsøgspersonen har vandladning. Blodprøver tages. Undersøgelsen afsluttes.

**Flowchart;**

**Endpoints:**

Primære:

- Glukose, aminosyre, og lipidmetabolisk arterio-venøs balance kvantificeret med tracer metodik

- Signalaktivering i muskel og fedtceller

Sekundære:

- Cytokin-afgift i plasma

**Blodprøver:**

I alt forventes tappet ca. 400 ml blod per undersøgelsesdag (2000 ml i alt i løbet af de 5 forsøgsdage), prøverne fraktioneres i 1 ml plasma og serum rør, og fryses til -80º C. Når forsøget er helt afsluttet foretages samlet analyse. Der oprettes forskningsbiobank.

**Metaboliske analyser:**

Der udtages blodprøver til analyse for: Na^+^, K^+^, creatinin, insulin, glukagon, katekolaminer, cortisol, IGF1, væksthormon, frie fede syrer*, glycerol*, glukose*, carbamid, aminosyre tracers*, palmitat tracer*. (*=A-V differencer).

**Fedtmetaboliske analyser:**

Der anvendes en tritieret palmitat-isotop som tracer. Ved at måle ændringer i plasma-palmitat specifik aktivitet (forholdet mellem palmitat-isotop og total palmitat) kan palmitat omsætningen bestemmes. Der anvendes kontinuerlig i.v. infusion af palmitat-isotopen [9,10^3^H]-palmitat 0,3 μCi/min i 2 x 60 minutter.

**Aminosyremetaboliske analyser:**

Der opsamles urin og bestemmes s-carbamid mhp vurdering af urinstofsyntesen (et udtryk for proteinnedbrydning).

Proteolyse og proteinsyntesehastigheden bestemmes desuden ved infusion af ^15^N-phenylalanin, ^15^N –Tyrosin og ^2^H_4_–Tyrosin.

*Aminosyre kinetik.* For at kunne udregne aminosyre kinetik anvendes i afdelingen tidligere beskrevne metoder: Qflux = *i*[(Ei/Ep) – 1]

hvor *i* er tracer infusionshastighed (mmol kg^–1^ h^–1^), og Ei og Ep er isotopberigelse (enrichment) i den infunderede tracer og i plasma.

For eksempel udregnes Phenylalanin balancer (PheBal) ved:

PheBal = (PheA – PheV) _ F

hvor PheA og PheV er phenylalanin koncentrationer i arterier og vener og F er blodgennemstrømning.

Regionale phenylalanin fluxe udregnes som følger:

Ra Phe = PheA [(PheEA/PheEV) – 1] _ F

hvor PheEA og PheEV er phenylalanin isotop berigelse i arterier og vener.

Rd kan nu bestemmes: Rd Phe = Phe Bal + Ra Phe.

**Indirekte kalorimetri**

Ved hjælp af en Deltatrac monitor kan man måle forsøgspersonernes iltforbrug og kuldioxid produktion, disse værdier kan lægges til grund for en udregning af en respiratorisk kvotient(RQ). Denne siger noget om forsøgspersonen forbrænder glukose eller fedt. Metoden er non-invasiv og kræver kun at forsøgspersonen ligger i en seng med en luftgennemstrømmet kuppel over hovedet.

**Immunologiske analyser:**

Blodprøver analyseres med Luminex® for cytokiner: IL-1, IL-2, IL-4, IL-6, IL-8, IL-10, IL-12, IFN-γ, TNF-α.

Der tages venøst fuldblod med henblik på fænotype karakteristik, adhæsionsmolekyler samt intracellulær cytokin produktion blandt de cirkulerende lymfocyt populationer i perifert blod ved hjælp af multicolor flow cytometri.

**Muskel biopsi:**

Der foretages i alt 2 muskel biopsier per forsøgsdag. Et område på lateralsiden af det ene lår desinficeres med jodsprit og der lægges lokalbedøvelse i to omgange med lidokain (10 mg/ml) svarende til hud, underhud og muskelfascie. Der laves mindre incision i hud og fascie med skalpel, og der udtages med Bergström biopsinål ca. 300 mg muskelvæv fra m. vastus lateralis. Muskel biopsien nedfryses umiddelbart i flydende kvælstof.

Undersøgelser på udtaget muskel væv vil omfatte insulins intracellulære signalveje (PI3 kinase, Akt mv.), aminosyre signaleringsveje og SOCS/STAT systemet: aktiveret STAT5, MAP-kinasernes aktivitet, SOCS mRNA, IGF-I mRNA, PI3-kinase aktivitet, AS160 fosforylering, NF-κB aktivering, samt bestemmelse af kvantitativt mRNA m.v.

**HÅNDTERING OG ARKIVERING AF DATA:**

Data opbevares i anonymiseret form. Data arkiveres på afdeling MEA, Århus Sygehus NBG. Data skal arkiveres i 15 år efter undersøgelsens afslutning.

**RISICI:**

Alle nålestik er forbundet med en meget lille risiko for betændelse ved indstiksstedet. De tagne muskelbiopsier er forbundet med lokal smerte/ømhed, efter vores erfaring få dage efter forsøget. Ved tegn på betændelse i huden eller dybere (rødme, varme, ømhed, hævelse) eller blot mistanke herom, skal du kontakte den projektansvarlige læge Nikolaj Fibiger Rittig (61714731) eller bagvagtslægen på afd. MEA (ringe til Århus Sygehus på 7845 0000 og bede omstillingsdamen om at kontakte medicinsk bagvagt på afdeling MEA (evt. bede om telefon 62067)).

Af de mange hundrede muskelbiopsier, der blev udført på vores afdeling, har vi stort set ikke oplevet nogen betydelige bivirkninger. Der skal dog nævnes et enkelt tilfælde af tab af muskelmassen i musklen, som biopsien blev taget fra. Dette er en forbigående komplikation og har ingen betydning for hverken stand og gang eller udøvelse af sport.

Endotoksin stammer fra bakteriers cellevæg, og bruges rutinemæssigt til at fremkalde en kontrolleret betændelsestilstand hos raske forsøgspersoner, således at sygdomsmekanismerne kan studeres. Hos forsøgspersoner som får større mængder endotoksin kan de forekomme influenza-symptomer med let ubehag, kulderystelser, muskelømhed, puls stigning, feber og hurtig vejrtrækning. Den lille dosis, vores forsøg er baseret på, er ikke vist at give disse symptomer.

Der er en mindre risiko for at blodsukkeret bliver lavt efter undersøgelsen, derfor får man et måltid. Såfremt du får symptomer herpå i form af hjertebanken, svedeture, ubehag eller rysten på hænder vil vi kontrollere blodsukkeret og behandle med sukkerindtag/indgift. Efter vores erfaring optræder der ikke lavt blodsukker efter 1 times observation, men for en sikkerheds skyld får du druesukker tabletter med hjem.

Der vil blive indgivet små mængder radioaktivt mærket fedt og sukker, det er nødvendigt for at kunne følge stofskiftet. Den samlede strålebelastning i løbet af undersøgelsesdagen svarer ca. til 15 % af den stråling man normalt modtager i løbet af et år (baggrundsstrålingen). Man kan teoretisk beregne den ekstra risiko ved strålingen til en 0,009 % forøget risiko for pådragelse af en kræft risiko i løbet af ens livstid. Alle de anvendte undersøgelsesmetoder er rutine på vort forskningslaboratorium.

Der kan i forbindelse med forsøget opstå uventede komplikationer.

**REKRUTTERING:**

Vil foregå ved hjælp af opslag på [www.forsøgsperson.dk](http://www.forsøgsperson.dk) , på opslagstavlerne på Århus Universitet og i de lokale aviser.

**KVALITETSSIKRING /KONTROL:**

Undersøgelsen følger GCP (Good Clinical Practice) retningslinjer.

Lægemiddelstyrelsen har godkendt anvendelse af endotoksin som et såkaldt ”værktøj” til induktion af inflammation.

Det tilkendegives hermed, at forsøget udføres i overensstemmelse med protokollen, gældende myndighedskrav og lovgivning foruden GCP retningslinjer. Undersøgelsens start og gennemførelse forudsætter således godkendelse fra den Videnskabsetiske Komité i Region Midtjylland, Lægemiddelstyrelsen forelægges protokollen. Undersøgelsen vil blive anmeldt til Datatilsynet.

Undersøgelsen vil blive udført i overensstemmelse med Helsinki deklaration.

Investigator giver de videnskabsetiske komitéer eller tilsvarende myndighed adgang til at kunne kontrollere relevante data. Projektet skal godkendes af den Videnskabsetiske Komité for Region Midtjylland, af Lægemiddelstyrelsen og Datatilsynet.

**ETISKE OVERVEJELSER:**

**Etiske overvejelser med hensyn til intervention:**

Eftersom sepsis, insulinresistens og proteintabet er centrale problemstillinger hos kritisk syge patienter og det stadigt er videnskabeligt uklart hvordan inflammation påvirker metabolismen hos mennesket, finder vi, at tort og svie hos forsøgspersonerne står mål med den mulige indsigt, forsøgsrækken ventes at bibringe.

Endotoksin indgivet i større doser kan give de inflammatoriske bivirkninger, hovedsageligt i form af influenza lignende symptomer, kvalme, opkastninger, feber, hovedpine, muskelømhed^8^ og i svære tilfælde sepsis og septisk chok^9,10^.

Når de gælder endotoksin har vi gode erfaringer i vores laboratorium, både ved indgivelse lokalt i benet, samt systemisk indgivelse til raske personer og patienter. De systemiske doser er baserede på tidligere studier, en kendt inflammations-model for kontinuerlig intravenøs endotoksin indgivelse^11^.

Samlet set forventes ikke at endotoksin vil give nogle bivirkninger, og i værste tilfælde vil det dreje sig om temperaturstigning og lette influenzalignende symptomer. Der er ikke beskrevet mere alvorlige bivirkninger, men forsøgspersonernes basale værdier vil blive monitoreret i løbet af studiet.

**Etiske overvejelser med hensyn til anvendte metoder:**

Den samlede strålebelastning er ca. 1.0 mSv (per forsøgsdag), hvilket svarer til ca. 15 % af den stråling man normalt modtager i løbet af et år (baggrundsstrålingen). Man kan teoretisk beregne den ekstra risiko ved strålingen til en 0,009 % forøget risiko for pådragelse af dødelig stråleinduceret kræft i løbet af ens livstid. En gennemsnitsdansker vil altså forøge sin risiko fra 25,00 % til 25,009 % per forsøgsdag.

Muskel biopsitagning med Bergström kanyle er en rutine på medicinsk laboratorium og i de mange tagne muskelbiopsier er der observeret et enkelt tilfælde af (reversibel) atrofi af m. vastus lat.

**SIKKERHEDSVURDERING:**

Inden selve undersøgelsesdagene vil der som anført blive foretaget en helbredsundersøgelse.

Der vil under hele forsøget være en læge og en bioanalytiker tilstede, der vil foretage en almenvurdering af forsøgspersonen og foretage måling af vitale parametre (blodtryk, puls, temperatur, blodsukker og bevidsthedsniveau).

Alvorlig hændelse defineres som følger: Ethvert medicinsk tilfælde uanset dosis som: Resulterer i død, er livstruende, medfører hospitalisering eller forlængelse af eksisterende hospitalisering, resulterer i vedvarende eller betydelig anomali eller betydelig invalidi­­­­tet/uarbejdsdygtighed eller giver en medfødt anomali/misdannelse.

Hvis der indtræffer alvorlig hændelse/bivirkning rapporteres disse på et skema til indberetning af alvorlig hændelse/bivirkning set i forbindelse med kliniske forsøg og andre hændelser/bivirkninger noteres i protokollen, der udfyldes for hver forsøgsperson. Ved alvorlige hændelser, som er en følge af projektet, vil den forsøgsansvarlige indberette de oplysninger, Videnskabsetisk Komité måtte anmode om. Hvis der optræder alvorlige hændelser/bivirkninger indsendes en liste til Videnskabsetisk Komité. Registreringen af bivirkninger ophører, når observationstiden slutter, idet det forventes, at den infunderede endotoksin dosis er udskilt af kroppen på det tidspunkt.

Komplikationer forventes at kunne registreres i forbindelse med ovennævnte lægelige objektive observationer og behandles på forsøgslaboratoriet, idet forsøget udføres på et højt specialiseret sygehus. I tilfælde af alvorlig hændelse/bivirkning stoppes forsøget for den enkelte øjeblikkeligt og eventuel påkrævet behandling institueres. Derefter vurderes det, om forsøget som sådant skal bringes til ophør. I tilfælde af hændelser/bivirkninger planlægges det at følge forsøgspersonen indtil symptomfrihed og/eller at tilstanden er stationær.

Alle bivirkninger/hændelser vil endvidere fremgå af den afsluttende rapport.

I henhold til lov om klage- og erstatningsadgang indenfor sundhedsvæsenet er deltagerne forsikret, hvis der skulle ske påvirkninger af helbredstilstand som følge af deltagelse i undersøgelsen.

**STATISTIK:**

Sammenligninger vil blive foretaget med standard statistiske metoder (t-test eller tilsvarende nonparametrisk test). Tidsserier og effekter af interventioner vil blive analyseret med analysis of variance (ANOVA) for gentagne målinger. P værdi under 0.05 antages som statistisk signifikant.

Styrkeberegninger vil ikke blive udført for alle effektmål, idet vi i sagens natur ikke har relevante værdier for alle parametre, men baseres på insulinsensitivitet i løbet af *clamp* (“M-værdi”).
 Antal N = 2 x f (α,β) x (σ/ δ)^2^
α er en risiko for type 1 fejl, β er en risiko for type 2 fejl, σ er SD og δ er en forskel, der kan ses. Vi har tidligere fundet en M-værdi med SD på 5 %, og β på 20 % giver f = 7.9. Hvis vi gerne vil se en forskel i M-værdi på 7 %, så er n = 2 x 7.9 x (5/7)2 = 8.01, dvs. N = 8.

Alle data anonymiseres og koden kendes kun af forsøgsansvarlig læge.

**RETNINGSLINIER FOR SKRIFTLIG OG MUNTLIG INFORMATION:**

Den mundtlige og skriftlige information gives i overensstemmelse med Forskningsstyrelsens Vejledning om information og samtykke ved inddragelse af forsøgspersoner i biomedicinske forskningsprojekter. Efter at de har læst opslaget, vil de potentielle forsøgspersoner rette henvendelse til investigator (via mail eller telefon), og der vil blive aftalt tid og sted for et møde, hvor de potentielle forsøgspersoner vil modtage mundtlig information. Der vil blive informeret om muligheden for at medbringe bisidder til samtalen. Den Centrale Videnskabsetiske Komités informationsfolder ”Forsøgspersonens rettigheder i et biomedicinsk forskningsprojekt” sammen med skriftlig deltager information tilsendes, således at der har været mulighed for gennemlæsning inden mundtlig information. Gennemlæsning af hele materialet vil aktivt blive anbefalet.

Den mundtlige information vil blive givet af læge Nikolaj Fibiger Rittig. Den mundtlige information om undersøgelsen vil foregå i enerum. Der vil være mulighed for at stille spørgsmål og for at kunne frasige sig viden om egen helbredstilstand.

Det vil blive understreget, at deltagelsen er frivillig og at tilsagn om deltagelse derfor kan trækkes tilbage på ethvert tidspunkt i forsøget, uden at dette vil påvirke læge-patientforholdet hverken aktuelt eller på et senere tidspunkt i forhold til Sundhedsvæsenet. Efter informationssamtalen vil der være mindst 1 døgns betænkningstid inden indhentelse af skriftlig samtykke på ”Standard samtykkeerklæring” tilføjet passage om forsøgspersonens samtykke til oprettelse af forskningsbiobank med opbevaring af biologisk materiale. Forundersøgelse vil eventuelt foregå efter mundtlig information, såfremt det skønnes mest praktisk.

**FINANSIERING:**

Projektet og lønudgifter finansieres af forskningsstøtte fra ”Keto projektet” med journalnummer 11-115840. Det er her Professor, Dr. med, overlæge Niels Møller som er initiativtager til det overordnede projekt.

Arla Foods group P/S har givet finansiel støtte til det overordnede ”Keto projekt” i form af 800.000 kr. som primært bruges på udvikling og produktion af de specifikke ernæringstilskud som gives under forsøget. Der er ingen tilknytning eller konfliktinteresser mellem støttegiver og forsøgsansvarlig.

Eventuelt ydereligere støtte til lønudgifter fra stipendiat Aarhus Universitet eller andre fonde som søges undervejs.

**KOMPENSATION:**

Forsøgspersoner vil modtage 1200 kr. pr. forsøgsdag. Det vil sige 6000 kr. for de 5 forsøgsdage

Det specificeres overfor deltagere, at godtgørelsen er skattepligtig. Forsøgspersoner vil blive udstyret med taxa bon, således at motion umiddelbart inden fremmøde på dagen undgås.

**PUBLIKATIONER:**

Disse kliniske studier forventes at resultere i mindst 4 publikationer i et internationalt peer-reviewed tidsskrift med Nikolaj Fibiger Rittig som førsteforfatter og Niels Møller som seniorforfatter.
Positive, negative og såvel inkonklusive resultater vil blive publiceret.

**REFFERENCER:**

1. Ziegler TR. Nutrition support in critical illness--bridging the evidence gap. *N Engl J Med.* *2011; 365: 562-4.*
2. Fearon KC. Cancer cachexia and fat-muscle physiology. *N Engl J Med. 2011; 365: 565-7.*
3. [Zudin Puthucheary](http://jp.physoc.org/search?author1=Zudin+Puthucheary&sortspec=date&submit=Submit)[^1^](http://jp.physoc.org/content/588/23/4641.long#target-1), [Hugh Montgomery](http://jp.physoc.org/search?author1=Hugh+Montgomery&sortspec=date&submit=Submit)[^2^](http://jp.physoc.org/content/588/23/4641.long#target-2), [John Moxham](http://jp.physoc.org/search?author1=John+Moxham&sortspec=date&submit=Submit)[^3^](http://jp.physoc.org/content/588/23/4641.long#target-3), [Stephen Harridge](http://jp.physoc.org/search?author1=Stephen+Harridge&sortspec=date&submit=Submit)[^4^](http://jp.physoc.org/content/588/23/4641.long#target-4)

and [Nicholas Hart](http://jp.physoc.org/search?author1=Nicholas+Hart&sortspec=date&submit=Submit)[^5^](http://jp.physoc.org/content/588/23/4641.long#target-5) Structure to function: muscle failure in critically ill patients*.* [*J Physiol.*](javascript:AL_get(this,%20'jour',%20'J%20Physiol.');) *2010 Dec 1; 588(Pt 23):4641-8.*

1. Weitzel LR, Sandoval PA, Mayles WJ, Wischmeyer PE. Performance-enhancing sports supplements: role in critical care. *Crit Care*

*Med. 2009 Oct; 37(10 Suppl):S400-9.*

1. Suryawan A, Jeyapalan AS, Orellana RA et al. Leucine stimulates protein synthesis in skeletal muscle of neonatal pigs by enhancing

mTORC1 activation. *Am J Physiol Endocrinol Metab* *2008; 295: E868-875.*

1. Gustafsson UO, Ljungqvist O. Perioperative nutritional management in digestive tract surgery. *Curr Opin Clin Nutr Metab Care.*

*2011; 14: 504-9.*

1. Andreasen AS, Krabbe KS, Krogh-Madsen R, Taudorf S, Pedersen BK, Møller K. Human endotoxemia as a model of systemic inflammation. *Curr Med Chem. 2008; 15: 1697-705. Review*
2. Vesali RF, Klaude M, Rooyackers O, Wernerman J: Amino acid metabolism in leg muscle after an endotoxin injection in healthy

volunteers. *Am J Physiol, Endocrinol Metab 2004, 288: E360-64.*

1. Tracey K, Beutler B, Lowry S, et al: Shock and tissue injury induced by recombinant human cachectin. *Science 1986, 234: 470-474*
2. Tracey K, Fong Y, Hesse D, et al: Anti-cachectin/TNF antibodies prevent septic shock during lethal bacteremia. *Nature 1987, 330:*

*662-664*

1. Taudorf S, Krabbe KS, Berg RM, Pedersen BK, Moller K: Human models of low-grade inflammation: bolus versus continuous infusion of endotoxin. *Clin Vaccine Immunol 2007, 14(3): 250-2551*
2. Cahill GF Jr. Fuel metabolism in starvation. *Annu Rev Nutr* *2006; 26: 1-22.*
